# WRN regulates pathway choice between classical and alternative non-homologous end joining

**DOI:** 10.1038/ncomms13785

**Published:** 2016-12-06

**Authors:** Raghavendra A. Shamanna, Huiming Lu, Jessica K. de Freitas, Jane Tian, Deborah L. Croteau, Vilhelm A. Bohr

**Affiliations:** 1 Laboratory of Molecular Gerontology, Biomedical Research Center, 251 Bayview Boulevard, National Institute on Aging, NIH, Baltimore, Maryland 21224, USA

## Abstract

Werner syndrome (WS) is an accelerated ageing disorder with genomic instability caused by WRN protein deficiency. Many features seen in WS can be explained by the diverse functions of WRN in DNA metabolism. However, the origin of the large genomic deletions and telomere fusions are not yet understood. Here, we report that WRN regulates the pathway choice between classical (c)- and alternative (alt)-nonhomologous end joining (NHEJ) during DNA double-strand break (DSB) repair. It promotes c-NHEJ via helicase and exonuclease activities and inhibits alt-NHEJ using non-enzymatic functions. When WRN is recruited to the DSBs it suppresses the recruitment of MRE11 and CtIP, and protects the DSBs from 5′ end resection. Moreover, knockdown of *Wrn*, alone or in combination with *Trf2* in mouse embryonic fibroblasts results in increased telomere fusions, which were ablated by *Ctip* knockdown. We show that WRN regulates alt-NHEJ and shields DSBs from MRE11/CtIP-mediated resection to prevent large deletions and telomere fusions.

## Abstract (web summary)

Werner Syndrome is an accelerated aging disorder marked by genome instability, large deletions and telomere fusions, hallmarks of aberrant DNA repair. Here the authors report a role for the WRN helicase in regulating the choice between classical and alternative non-homologous end-joning.

Werner Syndrome (WS) is an autosomal-recessive genetic disorder characterized by premature ageing and DNA repair defects because of mutations in the *WRN* gene^1^,^2^. Clinical manifestations in WS patients show a scheduled hierarchical deterioration of connective tissue and of the endocrine-metabolic system. Later, the immune and central nervous systems are affected, and there is an increased incidence and early onset of specific cancers^2^. Genomic instability is considered the major cause for the accelerated ageing in WS patients. Cells derived from WS patients are highly sensitive to DNA double-strand breaks (DSBs) and display variegated translocation mosaicism with chromosome aberrations^3^,^4^. WS cells and *Wrn* knockout mouse cells show genome instability, often with large deletions and telomere fusions^3^,^5^,^6^,^7^,^8^. However, it is unclear how WRN-deficiency leads to these biological consequences.

WRN is a RecQ family protein with helicase, strand annealing and exonuclease activities. WS cells and WRN-depleted cells show hypersensitivity to several types of DNA-damaging agents, indicating its role in DNA repair. WRN localizes to the sites of damaged DNA, interacts with several DNA repair proteins and participates in multiple DNA repair pathways including base excision DNA repair, non-homologous end-joining (NHEJ), homologous recombination (HR) and replication re-start after DNA damage^7^,^9^,^10^,^11^.

DSBs are highly toxic to cells and improperly repaired DSBs cause genome instability and cell death. In mammalian cells, DSBs are mainly repaired by NHEJ and HR. NHEJ occurs throughout the cell cycle and recent evidence suggests the existence of at least two sub-pathways, classical (c)-NHEJ and alternative (alt)-NHEJ. Previous work from our lab and others showed that WRN interacts functionally with multiple proteins in the c-NHEJ pathway including Ku70/80, DNA-dependent protein kinase catalytic subunit (DNA-PKcs), XRCC4 and DNA ligase IV (refs 4, 12, 13, 14). The Ku70/80 heterodimer, with its high DNA binding affinity, forms a stable complex with DNA-PKcs and initiates the DNA damage response signalling cascade for the NHEJ pathway^15^. The Ku70/80 complex interacts directly with WRN and stimulates its exonuclease activity^12^,^14^. DNA-PKcs, which gains robust kinase activity by interacting with DSB-bound Ku70/80, phosphorylates and regulates WRN's enzymatic activities^4^,^16^. Using its nuclease activity, WRN processes DNA ends to generate substrates suitable for ligation mediated by the XRCC4-DNA ligase IV complex^13^.

When core NHEJ proteins, Ku70/80 or ligase IV, are blocked or impaired, DSBs are channelled to the alt-NHEJ pathway^17^,^18^. Alt-NHEJ is distinguished from c-NHEJ by the participating proteins and by use of microhomology. Alt-NHEJ depends on several proteins that participate in HR; however, the pathway does not involve homologous sister chromatid formation, an obligate step in HR. MRE11, PARP1, carboxy-terminal binding protein (CtBP)-interacting protein (CtIP), DNA ligase I and DNA ligase III all promote alt-NHEJ (refs 19, 20, 21). During alt-NHEJ, MRE11 and PARP1 likely perform the DNA damage recognition, while CtIP and the MRN complex (MRE11, RAD50 and NBS1) process the broken ends by resection. Subsequently, the resected ends are ligated by DNA ligase I or ligase III (refs 19, 20, 21, 22, 23).

DNA repair by c-NHEJ is required for genome stability and suppression of translocations, and alt-NHEJ has been suggested to pose a particular threat to genome integrity^24^,^25^. The molecular mechanisms and the biological roles of the alt-NHEJ pathway is the subject of intense study. In the absence of c-NHEJ, alt-NHEJ is robust and acts as a backup DSB repair pathway^17^,^26^. Alt-NHEJ catalyses DSB repair resulting in chromosome translocations, deletions and fusions, which are considered detrimental to the cell^25^,^27^,^28^,^29^. However, alt-NHEJ is proposed to play a beneficial role during class switch recombination (CSR), an essential process that generates antibody isotypes^30^. During CSR, microhomologies present in the switch regions of DNA elements are recombined via alt-NHEJ (ref. 30). Further, alt-NHEJ is found to restore CSR defects by ∼50% in the absence of c-NHEJ (ref. 31). Interestingly, inhibition of WRN's helicase activity in 53BP1 or H2AX null cells increased class switch recombination^32^. Although WRN interacts with several proteins that promote alt-NHEJ (refs 9, 33, 34), its involvement in alt-NHEJ or the pathway choice is yet to be determined.

Here we have performed *in vitro* and *in vivo* NHEJ assays in HeLa and U2OS cell lines, as well as in lymphoblast cells derived from WS patients to investigate the role of WRN in NHEJ and its sub-pathways. Knockdown of WRN significantly reduced total-NHEJ and increased 5′ end resection and alt-NHEJ. To delineate the molecular mechanism involved in the pathway choice, we tested the recruitment of MRE11 and CtIP and found that WRN suppresses the recruitment of both proteins to laser-induced DSBs. Furthermore, Wrn knockdown in mouse embryonic fibroblasts (MEFs) increased telomere fusions, which was suppressed by CtIP knockdown. Altogether these results suggest that WRN regulates pathway choice between c-NHEJ and alt-NHEJ, and that WRN deficiency enhances genomic instability through destabilizing NHEJ.

## Results

### WRN regulates NHEJ repair *in vitro*

**Figure 1:**
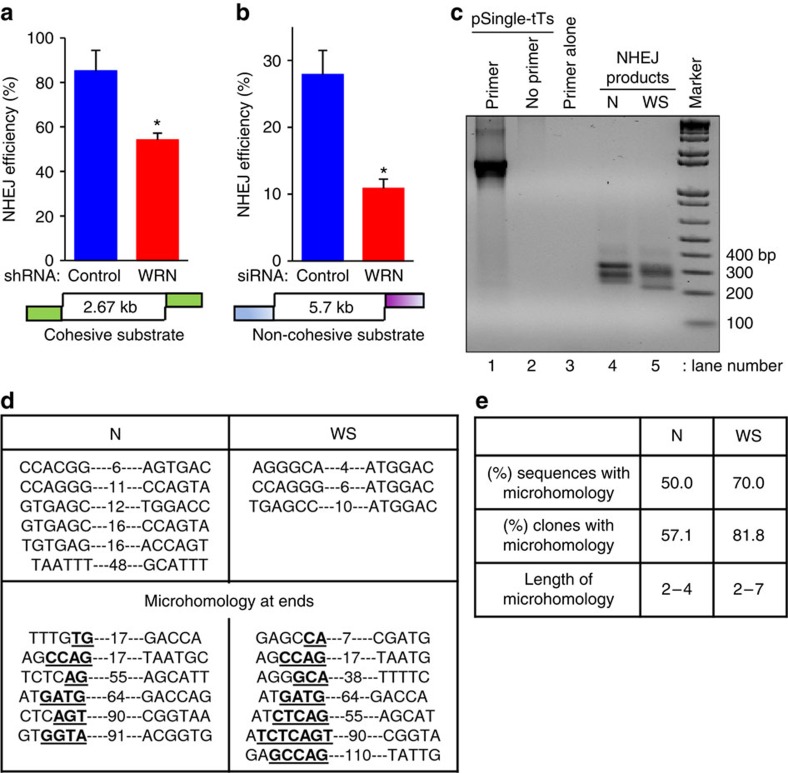
WRN deficiency reduces NHEJ. (**a**) Graph indicating *in vitro* NHEJ efficiency of control and WRN shRNA expression HeLa cell extracts with 2.67 kb cohesive-end (*Sal*I-linearized) pUC18 DNA substrate. (**b**) NHEJ efficiency of U2OS cell extracts on 5.7 kb non-cohesive DNA substrate derived from *Bst*XI-digested pSingle-tTS-shRNA plasmid. Graphs represent end-joining efficiency from three independent experiments. *, *P* value <0.05. Ends of DNA substrate are colour coded to indicate cohesive (green) and non-cohesive (discontinuous blue and pink) ends. (**c**) Agarose gel showing PCR amplified products of uncut pSingle-tTS-shRNA plasmid and DNA isolated from NHEJ reactions shown in Supplementary figure 1B. Amplified bands from end joined regions can be seen in lanes 4 and 5. (**d**) Junctional sequence analysis of *in vitro* NHEJ products. DNA sequences of end-joined regions cloned from PCR amplified regions of NHEJ products shown in **c**. Numbers flanked by dashes represent number of nucleotides deleted after end joining, nucleotide sequences in bold and underlined represent microhomology sequence used for end-joining. (**e**) Table summarizing characteristics of end-joined sequences. N; SRY110010 normal lymphoblasts, WS; SRY110011 Werner syndrome patient lymphoblasts.

To investigate the role of WRN in c-NHEJ and alt-NHEJ, we performed *in vitro* and *in vivo* NHEJ assays^17^,^35^,^36^,^37^. *In vitro* NHEJ was performed with DNA substrates containing cohesive and non-cohesive ends and cellular extracts prepared from HeLa or U2OS cells treated with control and WRN short hairpin (sh) RNA or with small interfering (si) RNA (Supplementary Fig. 1A). Knockdown of WRN in HeLa cells reduced *in vitro* end-joining efficiency by ∼36% compared with control cells (Fig. 1a). In U2OS cells, WRN knockdown inhibited total NHEJ by ∼61% on non-cohesive substrates (Fig. 1b). These results suggest that WRN promotes NHEJ *in vitro*.

To characterize the reaction products and to ascertain how much microhomology-mediated repair had taken place, the end joined regions were sequenced. In these reactions, the DNA substrate had non-compatible ends and the extracts were prepared from normal and WS lymphoblasts (Supplementary Fig. 1B). The end-joined regions were amplified by polymerase chain reaction (PCR) (Fig. 1c) and sequenced after cloning the amplified products into a pUC18 plasmid. PCR amplified products displayed differential mobility patterns in agarose gels indicating differences in end processing during NHEJ reactions in the presence or absence of WRN (Fig. 1c). Further, WRN proficient and WRN deficient cells displayed differential microhomology usage during the end-joining reaction (Fig. 1d,e). Interestingly, WRN deficiency increased the microhomology usage by ∼40%. The length of the microhomology was 2-4 nucleotides in the presence of WRN and 2-7 nucleotides in the absence of WRN. These results suggest that WRN inhibits microhomology-mediated end-joining, a feature of alt-NHEJ.

Since WRN physically and functionally interacts with many proteins participating in c-NHEJ and alt-NHEJ, we tested whether loss of WRN changes the levels of major proteins involved in these pathways. Knockdown of WRN with shRNA in HeLa cells or with siRNA in U2OS cells did not alter the expression of proteins associated with c-NHEJ (DNA-PKcs, Ku70, Ku80, 53BP1, DNA ligase IV, Artemis and XLF) or alt-NHEJ (CtIP, PARP1, XRCC1, DNA ligase I and DNA ligase III) (Supplementary Fig. 1A). We also examined the expression of these proteins in lymphoblasts and fibroblasts derived from WS patients. Similar to transient WRN-knockdown cells, there was no significant differences in the expression of these proteins in WS cells compared with normal cells (Supplementary Fig. 1A). Together these results suggest that WRN deficiency reduces NHEJ efficiency without affecting the expression of major proteins involved in the NHEJ pathway.

### WRN promotes c-NHEJ and inhibits alt-NHEJ *in vivo*

**Figure 2:**
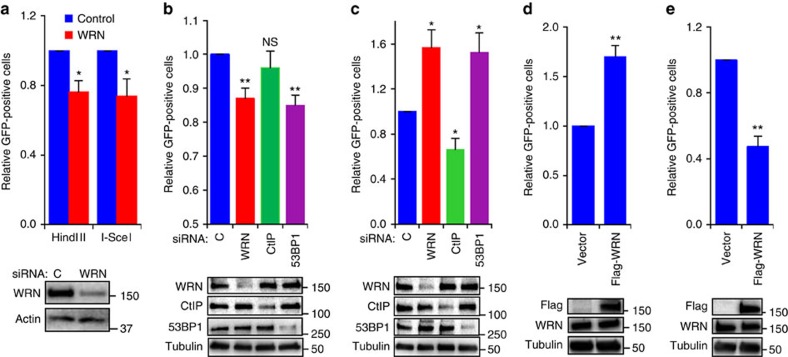
WRN promotes c-NHEJ and inhibits alt-NHEJ. (**a**) pEGFP-Pem1-Ad2 reporter based NHEJ efficiency in siRNA transfected U2OS cells. Control and WRN knockdown cells were transfected with *Hin*dIII (cohesive ends) or *I-Sce*I enzyme digested (non-cohesive ends) pEGFP-Pem1-Ad2 along with DsRed and analysed for GFP and DsRed expressing cells by flow cytometry. (**b**) Total NHEJ efficiency in siRNA transfected EJ5 cells. (**c**) Alt-NHEJ efficiency in siRNA transfected EJ2 cells. (**d**) Expression of WRN promotes NHEJ. (**e**) WRN expression suppresses alt-NHEJ. DSBs in EJ2 and EJ5 cells were induced by transfecting *I-Sce*I expression plasmid. Graph represents relative repair efficiency as measured by GFP-positive cells normalized to DsRed expression. Immunoblots represent protein expression levels. Error bars represent SEM from three independent experiments. *P* value, *, <0.05; **, <0.01; NS, not significant.

To corroborate our *in vitro* end-joining results on NHEJ and the microhomology usage, we examined the role of WRN in *in vivo* NHEJ using green fluorescence protein (GFP) reporter based assays. We utilized the well-established pEGFP-Pem1-Ad2 and EJ5 reporter systems to measure total NHEJ (c-NHEJ plus alt-NHEJ)^36^,^37^, and the EJ2 reporter system to measure alt-NHEJ activities^37^. The EGFP gene in pEGFP-Pem1-Ad2 is disrupted with a stuffer sequence containing Pem1 intron and adenoviral exon 2 (Supplementary Fig. 2A) which requires creation of DSBs by either *Hin*dIII or *I-Sc*eI restriction enzymes followed by repair of DSBs via NHEJ for the expression of GFP protein. We transfected either *Hin*dIII or *I-Sc*eI digested pEGFP-Pem1-Ad2 plasmid along with DsRed-Express-C1 into control and WRN knockdown U2OS cells to measure end-joining efficiency. NHEJ-dependent repair of *EGFP* was detected by fluorescence-activated cell sorting (FACS) and the data were normalized to DsRed expression. Knockdown of WRN reduced the end-joining efficiency of *Hin*dIII or *I-Sc*eI digested pEGFP-Pem1-Ad2 reporter constructs by ∼25% compared with control cells (Fig. 2a), suggesting that WRN promotes NHEJ.

We also performed NHEJ assays with EJ2- and EJ5-GFP reporter cassettes (Supplementary figure 2B and 2C) stably integrated into U2OS cells^37^,^38^. Expression of GFP from the EJ5 reporter cassette, which reflects a composite of c-NHEJ and alt-NHEJ, is observed only after expression of *I-Sce*I and DSB repair^38^. Knockdown of WRN reduced GFP expression from the EJ5 cassette by ∼13% compared with control siRNA transfected cells (Fig. 2b). Similar to WRN, knockdown of 53BP1 reduced the GFP-positive cell population by ∼15%; however, knockdown of CtIP, which does not participates in c-NHEJ, did not significantly alter the GFP-positive cell population. These results suggest that both WRN and 53BP1 promote *in vivo* NHEJ.

Next, to test whether alt-NHEJ is impacted by loss of WRN we measured GFP expression in the EJ2-U2OS cell line, which specifically measures alt-NHEJ repair after *I-Sce*I expression and depends on usage of an eight nucleotide microhomology sequence^38^. Interestingly, loss of WRN enhanced alt-NHEJ activity by ∼57% (Fig. 2c). Likewise knockdown of 53BP1, which inhibits resection and alt-NHEJ^39^, increased the GFP expression to the same level as WRN depletion (Fig. 2c). As expected, and consistent with a previous report^38^, knockdown of CtIP reduced alt-NHEJ in these cells. Ectopic expression of 3xFlag-WRN in EJ5 increased the GFP-positive cells by ∼70% (Fig. 2d), while the expression in EJ2 suppressed the GFP-positive cells by 53% (Fig. 2e). Together these results suggest that WRN promotes c-NHEJ and inhibits alt-NHEJ.

This is the first direct demonstration that WRN plays a role in c- and alt-NHEJ *in vivo*.

### Catalytic activities of WRN in c-NHEJ and alt-NHEJ

**Figure 3:**
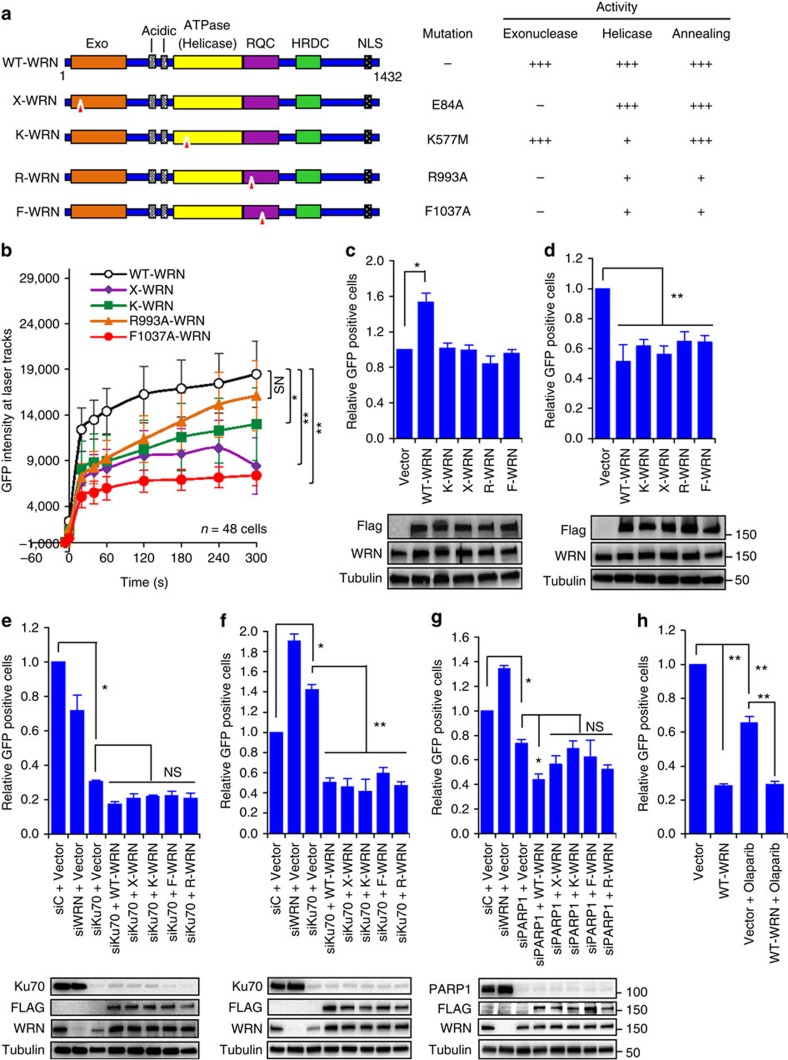
Enzymatic and non-enzymatic functions of WRN in c-NHEJ and alt-NHEJ. (**a**) Linear structure of WT and WRN mutants along with functional properties. Exo, exonuclease; +++, high activity; +, low activity; −, no activity. Triangles in red represent mutation position. (**b**) Recruitment kinetics of WT and WRN mutants to DSBs. U2OS cells expressing c-terminal GFP tagged WRN constructs were microirradiated and recruitment of GFP-tagged protein to DSBs were calculated. (**c**) Helicase and exonuclease activities of WRN are required for c-NHEJ. EJ5 cells were transfected with 3xFlag tagged WRN constructs along with *I-Sce*I and pDsRed-Express-C1. Relative NHEJ efficiency was measured based on GFP and DsRed expressing cells. (**d**) Enzymatic functions of WRN are not required for inhibiting alt-NHEJ. EJ2 cells were transfected with WRN constructs and end joining efficiency was analysed as in **c**. (**e**) WRN fails to stimulate c-NHEJ in Ku70 knockdown cells. 24 h post-siRNA transfection, EJ5 cells were co-transfected with plasmids expressing *I-Sce*I, DsRed and flag-tagged WRN constructs. Relative NHEJ efficiency was measured as in **c**. (**f**) WRN inhibits alt-NHEJ in Ku depleted cells. (**g**) WRN and PARP1 inhibit alt-NHEJ. 24 h post-siRNA transfection, EJ2 cells (Fig. 3f,g) were co-transfected with plasmids expressing *I-Sce*I, DsRed and flag-tagged WRN constructs, and relative NHEJ efficiency was measured as in **d**. (**h**) WRN inhibits alt-NHEJ in olaparib treated cells. Plasmid transfected cells were treated with olaparib for 4 days and then alt-NHEJ efficiency was measured. Error bars represent SEM from three independent experiments. *P* value, *, <0.05; **, <0.01; NS, not significant.

Since we identified that WRN differentially regulates c-NHEJ and alt-NHEJ, we next assessed the importance of WRN's exonuclease and helicase functions in these pathways (Fig. 3). The *In vitro* helicase, exonuclease and DNA annealing activities of wild type (WT), exonuclease-dead E87A (X-WRN), helicase-dead K577M (K-WRN) or RQC domain mutants (R993A-WRN, F1037A-WRN)^40^ are summarized in Fig. 3a. GFP-tagged WT and mutants were expressed in U2OS cells and analysed for their recruitment to laser-induced DSBs. All WRN mutants recruited to DSBs with less efficiency than WT-WRN (Fig. 3b). Helicase-dead and exonuclease-dead WRN displayed similar recruitment kinetics. Although R993A and F1037A mutants lack DNA binding activity *in vitro*^40^, they were recruited to DSBs, however the recruitment was severely reduced in the case of F1037A-WRN (Fig. 3b). To test the effect of these mutations on c-NHEJ and alt-NHEJ, EJ5 and EJ2 cells were transfected with pCMV-tag4 plasmids carrying 3xFlag-tagged WT-WRN, X-WRN, K-WRN, R993A-WRN or F1037A-WRN (Fig. 3c,d). WT-WRN stimulated the NHEJ activity with the EJ5 reporter by ∼53% (Fig. 3c). However, all mutants failed to enhance the NHEJ activity suggesting the importance of the catalytic activities of WRN in c-NHEJ (Fig. 3c). On the other hand both WT and mutants inhibited the alt-NHEJ mediated repair activity to a similar extent in EJ2 cells (Fig. 3d). Taken together these results suggest that WRN stimulates c-NHEJ with its helicase and exonuclease catalytic activities and that these catalytic activities are not required for inhibiting alt-NHEJ.

Since Ku70 and PARP1 are known to be important for c-NHEJ and alt-NHEJ we tested the role of WRN in regulating c-NHEJ and alt-NHEJ pathways under Ku70 and PARP1 deficient conditions. Knockdown of Ku70 in EJ5 cells reduced GFP-positive cells by ∼70% compared with control cells demonstrating the inhibition of c-NHEJ events (Fig. 3e). Ectopic expression of WT or mutant WRN constructs in Ku70 knockdown cells did not significantly alter the GFP-positive cell population (Fig. 3e), indicating that Ku is required for c-NHEJ and that WRN fails to stimulate c-NHEJ in the absence of Ku. To characterize the importance of WRN in regulating alt-NHEJ in the absence of Ku, EJ2 cells were transfected with Ku70 siRNA and with WT and mutant WRN plasmids (Fig. 3f). Similar to WRN, knockdown of Ku70 in EJ2 cells increased the GFP-positive cell population; however, ectopic expression of either WT or mutant WRN in Ku70 knockdown cells suppressed GFP-positive cell population. These results demonstrate that WRN suppresses alt-NHEJ activity under Ku-deficient conditions.

Previous findings suggest that PARP1 activity is important for alt-NHEJ^38^. Our results show that knockdown of PARP1 (Fig. 3g) or inhibition of PARP1 activity by olaparib (Fig. 3h) reduces alt-NHEJ-mediated DNA repair events in EJ2 cells. Interestingly expression of WT-WRN in PARP1 knockdown cells significantly increased inhibitory effect on alt-NHEJ; however, expression of mutant WRN constructs did not show such increase in alt-NHEJ (Fig. 3g). Treatment of cells with olaparib, which inhibits PARP1 activity, suppressed the alt-NHEJ mediated repair events by ∼33% compared with control cells (Fig. 3h). Ectopic expression of 3xFlag-WT-WRN alone or together with olaparib treatment suppressed the alt-NHEJ by ∼70% (Fig. 3h).

Taken together these results suggest that WRN efficiently suppresses alt-NHEJ activity in Ku70-deficient and PARP1-deficient cells.

### WRN suppresses recruitment of MRE11 and CtIP to DSBs

**Figure 4:**
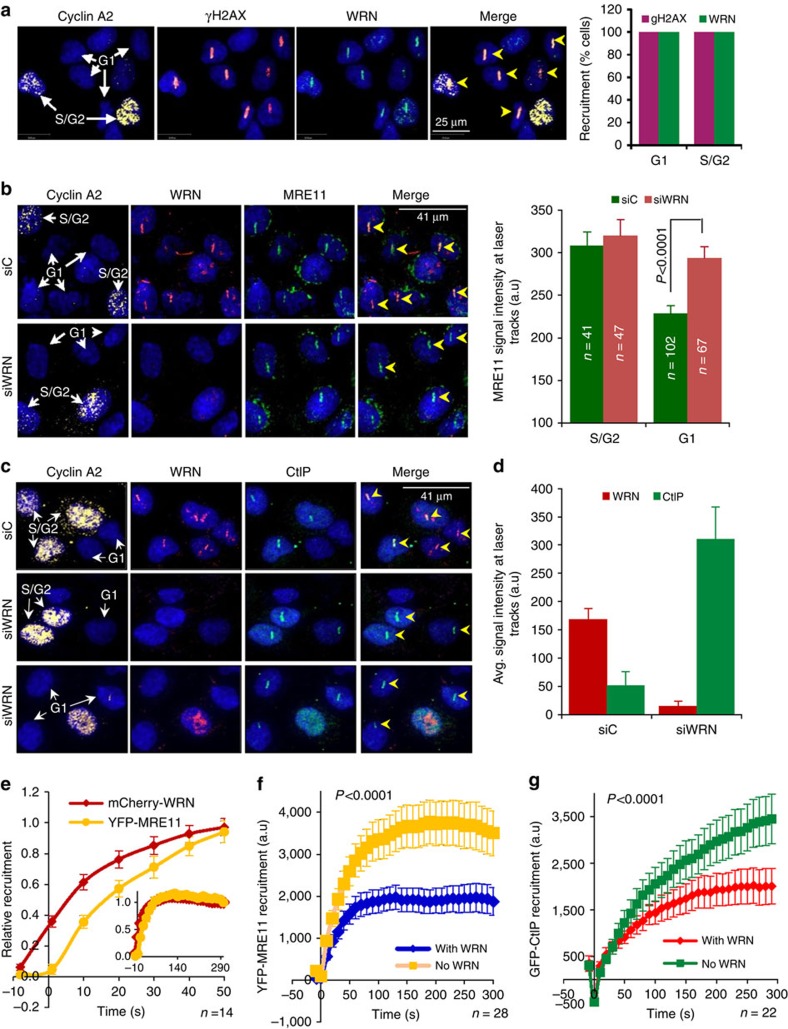
WRN inhibits the recruitment of MRE11 and CtIP to DSBs. (**a**) Confocal microphotographs showing cell cycle independent recruitment of WRN to laser-induced DSBs in U2OS cells. Graph represents data from >200 cells. gH2AX; gamma H2AX. (**b**) WRN depletion increases MRE11 recruitment to DSBs in G1 cells. siRNA transfected U2OS cells were microirradiated and immunostained for cyclin A2, WRN and MRE11. Graph indicates MRE11 recruitment to laser-induced DSBs in control and WRN siRNA transfected S/G2 and G1 cells. n; number of cells. (**c**) WRN knockdown promotes CtIP recruitment to laser-induced DSBs. Control (siC) and WRN knockdown U2OS cells were microirradiated to generate DSBs and immunostained with WRN, CtIP and cyclin A2 antibodies. Cyclin A2 positive cells indicate cells in S/G2 phase. (**d**) Quantitation of CtIP and WRN signals at DSBs as seen in panel E. *n*=26 cells per set (**e**) Recruitment kinetics of mCherry-WRN and YFP-MRE11 to laser-induced DSBs in U2OS cells. *n*=14 cells (**f**) Real-time recruitment of YFP-MRE11 to laser-induced DSBs in WS cells, AG11395, expressing no WRN (vector control) and mCherry-WRN. *n*=28 cells (**g**) Recruitment of GFP-CtIP to laser-induced DSBs in AG11395 WS cells transfected with or without mCherry WRN. *n*=22 cells. Yellow arrow heads in panel A, B and C indicate laser-induced DSB tracks. a.u., arbitrary unit.

HR and alt-NHEJ share the same initial DNA end resection steps in the repair of DSBs^22^, and it has been suggested that resection mediated by CtIP is a characteristic feature of alt-NHEJ^25^,^28^. To delineate the mechanism by which WRN inhibits alt-NHEJ, we first investigated the recruitment of MRE11 and CtIP to laser-induced DSBs in the absence and presence of WRN (Fig. 4). WRN was recruited to DSBs during all phases of the cell cycle (Fig. 4a). MRE11 was recruited to DSBs irrespective of the cell cycle (Fig. 4b, top panel), however, its recruitment was enhanced in WRN knockdown G1 cells (Fig. 4b). Since MRE11 physically and functionally interacts with CtIP promoting DSB resection^41^, we tested the recruitment of CtIP to DSBs. CtIP's resection activity has been found to be critical for DSB repair in S/G2 as well as G0/G1 cells^41^,^42^. In line with Sartori et al, 2007^41^, we found that CtIP recruits in the S/G2 phase of the cells in control siRNA transfected U2OS cells (Fig. 4c). Interestingly, CtIP recruited to DSBs in both S/G2 and G1 cells after WRN knockdown (Fig. 4c). Quantitation of fluorescence signals at laser-induced DSBs suggested that WRN depletion enhanced CtIP's recruitment by ∼6-fold (Fig. 4d).

To study the recruitment kinetics of WRN and MRE11, U2OS cells were co-transfected with mCherry-WRN and YFP-MRE11. Live-cell confocal imaging indicated that both mCherry-WRN and YFP-MRE11 recruit robustly to the DSB tracks, however, WRN was recruited prior to the recruitment of MRE11 (Fig. 4e, Supplementary fig. 3A, Supplementary Movie 1). Next, the effect of WRN on the recruitment of MRE11 was studied by expressing YFP-MRE11 along with mCherry-WRN or with control plasmid in WS patient fibroblasts, AG11395, which lack WRN. MRE11 was recruited to DSBs in WS cells in the presence or absence of WRN; however, the recruitment was significantly reduced in the presence of WRN (Fig. 4f, Supplementary fig. 3B, Supplementary Movie 2 and 3). To measure the real-time recruitment of CtIP, WS patient fibroblasts were transiently transfected with GFP-CtIP and mCherry-WRN (Fig. 4g, Supplementary fig. 3C, Supplementary Movie 4 and 5). WRN inhibited the recruitment of GFP-CtIP to the DSBs in these cells. Interestingly, knockdown of WRN had no significant effect on the recruitment of DNA ligase I (Supplementary fig. 4A) or ligase III (Supplementary fig. 4B), which are known to play roles in alt-NHEJ. Based on the real-time recruitment data, T1/2 for WRN, MRE11 and CtIP is ∼10, ∼20 and ∼100 s, respectively. The rate of MRE11 recruitment remained similar in the presence and absence of WRN (Supplementary Fig. 3D). Interestingly, CtIP displayed significantly different recruitment kinetics in the presence and absence of WRN. CtIP recruited to microirradiation-induced DSBs with a T1/2 of ∼100 s and ∼50 s in the absence and presence of WRN, respectively (Supplementary fig. 3E). Together, these results suggest that WRN suppresses the recruitment of MRE11 and CtIP to DSBs, with potential inhibitory effects on DNA end resection.

### WRN deficiency enhances resection at DSBs

**Figure 5:**
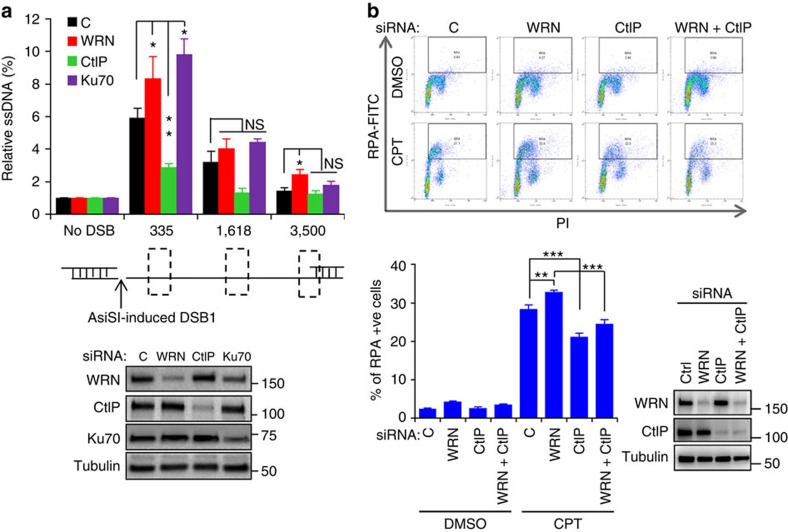
Deficiency of WRN enhances resection at DSBs. (**a**) Quantitation of 5′ end resection in WRN knockdown AID-DIvA cells. Bar graph showing the extent of ssDNA generated by end resection at *Asi*SI-induced DSBs. Three days post siRNA-transfection, cells were treated with 4-OHT for 4 h and quantitative PCR was performed to amplify the ssDNA at a specific DSB site. Immunoblots indicate the protein knockdown levels in siRNA transfected AID-DIvA cells. Cartoon below the bar graph represent the DSB site on chromosome 1 along with regions (dotted boxes) used to amplify the ssDNA generated from resection. Error bars represent SEM from four independent biological experiments. (**b**) ssDNA-bound RPA in WRN knockdown U2OS cells. Control, WRN and CtIP depleted cells treated with DMSO or camptothecin (CPT) were detergent extracted prior to fixation and staining with RPA and propodeum iodide (PI) and analysed by flow cytometry. Representative dot plots of RPA and PI stained cells are shown. Graph represents ssDNA-bound RPA from three biological experiments. Error bars indicate standard deviation. *P* value, *, <0.05; **, <0.01; ***, <0.001; NS, not significant. Immunoblots indicate protein levels in siRNA transfected cells.

CtIP in association with MRN regulates the resection during HR as well as during alt-NHEJ^22^, and thus we quantified 5′ end resection using AID-DIvA cells. These modified U2OS cells express *Asi*SI endonuclease tagged with hemagglutinin (HA)-oestrogen receptor ligand binding domain (ER), HA-ER-*Asi*SI, which translocates to the nucleus after tamoxifen (4-OHT) treatment and induce DSBs in genomic DNA^43^ (Supplementary figure 5). The extent of 5′ end resection, which produces ssDNA at a known DSB on chromosome 1 was scored by quantitative PCR as described before^44^. As expected, Ku70 knockdown increased the resection whereas CtIP knockdown reduced the resection (Fig. 5a). If WRN knockdown increases alt-NHEJ, as observed in Figure 2c, we would expect greater resection after WRN loss. Supporting that WRN has an inhibitory role, knockdown of WRN increased 5′ end resection in AID-DIvA cells and the resection was similar to that produced by Ku70 knockdown (Fig. 5a). WRN knockdown increased DNA resection by ∼40%, ∼25% and ∼71% at 335, 1618 and 3500, nt, respectively, from the DSB (Fig.5a). Interestingly, simultaneous knockdown of WRN and CtIP inhibited DSB resection suggesting that CtIP promotes resection in the absence of WRN (Supplementary fig. 6).

To measure the resection at random positions in genomic DNA, we employed a high-throughput flow cytometry-based assay, which quantifies ssDNA bound by RPA32^38^,^45^. This assay measures resection at DSBs and at stalled replication forks induced by camptothecin (CPT). Chromatin bound RPA32 in control and WRN knockdown U2OS cells treated with DMSO or CPT (1 μM) for 1 h was immunostained and detected by flow cytometry. After CPT treatment, ∼28% control and ∼33% WRN knockdown cells were positive for chromatin-bound RPA, indicating ∼18% increase in resection in WRN knockdown cells (Fig. 5b). Knockdown of CtIP significantly reduced the chromatin-bound RPA cell population and interestingly, knockdown of CtIP together with WRN significantly reduced the chromatin-bound RPA positive cells indicating that CtIP promotes resection in the WRN depleted cells (Fig. 5b). Together these results suggest that WRN protects the DSBs by limiting resection.

### CtIP is required for telomere fusions in WRN-depleted cells

**Figure 6:**
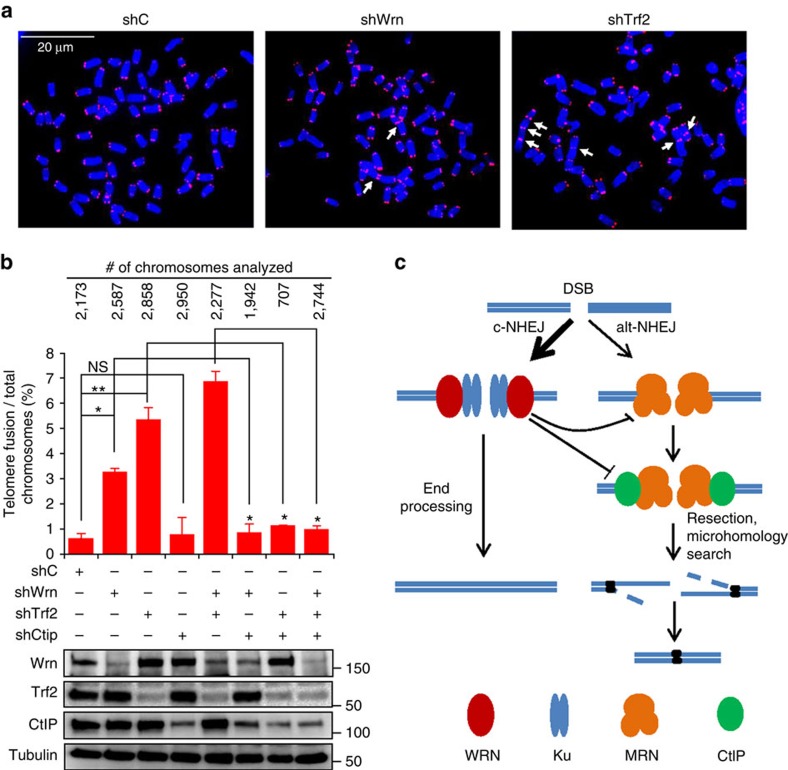
CtIP mediates telomere fusions in WRN-deficient MEFs. (**a**) Metaphase chromosomes from MEFs infected with lentivirus carrying control, Wrn and Trf2 shRNA. Ten days post-puromycin selection, cells were arrested in mitosis with colcemid and telomeres were detected by hybridizing the chromosomes with Cy3-conjugated (TTAGGG)_3_ probe (red). DNA was counterstained with DAPI (blue). White arrows indicate fused chromosomes. (**b**) Frequency of fused chromosomes in MEFs infected with lentivirus carrying shRNA. Mitotic chromosomes with telomere fusions were analysed and represented as per cent chromosomes with fused telomeres. Error bars represent standard deviation from two biological experiments. Immunoblots indicate expression levels of proteins in MEFs infected with lentivirus. (**c**) Cartoon showing WRN regulated c-NHEJ and alt-NHEJ. c-NHEJ is the major DSB pathway and is represented by thick arrow. WRN promotes c-NHEJ and inhibits alt-NHEJ by suppressing the recruitment and functions of MRE11 and CtIP. MRE11 and CtIP mediate DSB resection to promote alt-NHEJ, which results in large deletions and telomere fusions. Error bars indicate s.e.m. *P* value, *, <0.05; **, <0.01; NS, not significant.

Recent reports suggest that chromosomal translocation, class switch recombination and telomere fusions are mediated by CtIP and the alt-NHEJ pathway^25^,^28^,^30^. It is well documented that WRN deficiency induces telomere fusions^5^,^6^, however, the underlying mechanism is not understood. Therefore, we next asked whether CtIP promotes telomere fusions in WRN-deficient cells. Metaphase spreads were prepared from SV40-transformed MEFs, CRL-2037, knocked down with control shRNA (shC), shWrn, shTrf2 or shCtip, alone or in combination, and hybridized with telomere specific PNA probes. Chromosomes with fused telomeres are shown for Wrn and Trf2 knockdown cells (Fig. 6a). Quantitation of the fused chromosomes indicated that Wrn and Trf2 knockdown resulted in ∼3.4% and ∼5.4% of chromosomes showing telomere fusions, respectively (Fig. 6b). In contrast, knockdown of CtIP showed no change in telomere fusions (0.8% versus 0.6% for controls). MEFs knocked down for both Wrn and Trf2 displayed ∼6.9% telomere fusions. Knockdown of CtIP together with either Wrn and/or Trf2 eliminated the observed increase in telomere fusions, suggesting that CtIP regulates telomere fusions in the absence of Wrn and Trf2 (Fig. 6b). Taken together these results suggest that WRN suppresses alt-NHEJ and CtIP-mediated telomere fusions.

## Discussion

WS patients display chromosome aberrations with increased translocations, deletions, and telomere fusions^3^,^5^,^6^,^7^,^11^,^46^. However, the precise mechanism(s) that contributes to genome instability in WS and WRN-deficient cells remains unclear. Here, we identified a crucial role of WRN in regulating the pathway choice between c-NHEJ and alt-NHEJ. Using *in vitro* and *in vivo* assays, we found that WRN promotes c-NHEJ with its helicase and nuclease functions, and inhibits alt-NHEJ in a non-catalytic manner by suppressing MRE11 and CtIP. WRN deficiency increased resection at DSBs and induced telomere fusions via the alt-NHEJ pathway. These findings elucidate the molecular mechanisms underlying the increased telomere fusion and genome instability in WS cells.

DSBs generated by various exogenous and endogenous DNA damaging agents are predominantly repaired by HR, c-NHEJ and alt-NHEJ. WRN interacts with several proteins involved in these repair pathways; however, the mechanisms by which WRN regulates these repair pathways unclear. Loss of WRN causes a mild inhibition of HR suggesting that WRN may only play a minor role in HR^11^,^47^. In this study, we found that WRN regulates c-NHEJ and alt-NHEJ. In the absence of c-NHEJ, alt-NHEJ acts as the backup DNA repair pathway and is relatively robust^17^,^18^,^31^. WRN deficiency significantly reduced DNA repair with EJ5 and pEGFP-Pem1-Ad2 reporter cassettes, and ectopic expression of WRN stimulated the DSB repair by ∼70% with the EJ5 reporter cassette. Since alt-NHEJ shows strong bias towards microhomology-mediated end joining, and pEGFP-Pem1-Ad2 and EJ5 reporter cassettes are unable to distinguish DSB repair mediated by c-NHEJ and alt-NHEJ pathways, we measured alt-NHEJ events using the EJ2 reporter system. Knockdown of WRN in alt-NHEJ reporter cells increased the DSB repair by ∼57% while ectopic expression of WRN reduced the repair by about the same ∼53%. Knockdown of WRN in U2OS and HeLa cells did not alter the levels of major proteins associated with c-NHEJ and alt-NHEJ suggesting that the observed effects on NHEJ are mainly because of lack of WRN. From these studies it is evident that WRN suppresses alt-NHEJ and promotes c-NHEJ for DSB repair.

WRN is a multifunctional protein with four catalytic activities. The amino terminus contains the 3′-5′ exonuclease, while the central part of the protein harbours the DNA-dependent ATPase, 3′-5′ helicase and annealing activity^1^,^10^. WRN exonuclease and helicase activities are found to be important for DNA repair^11^,^48^. Here we find that mutations in WRN's nuclease domain (E84A), helicase domain (K577M), or DNA binding domain (R993A, F1037A) aborts the stimulatory effect of the protein on c-NHEJ. However, these mutations sustained the inhibitory effect on alt-NHEJ suggesting that the enzymatic functions of WRN are required to promote c-NHEJ but not required to inhibit alt-NHEJ. WRN mediates its helicase and exonuclease activity in 3′-5′ polarity^49^ and interestingly DSBs generated by *I-sce*I, *AsiS*I, *Sal*I and *Bst*XI used in the end-joining assays are characterized with 3′ overhangs. The inability of helicase-dead and exonuclease-dead WRN to stimulate c-NHEJ suggests that the catalytic activity of WRN is involved in processing the DSBs during end-joining. WRN's inhibitory effect on alt-NHEJ could be because of the physical presence of WRN at DSBs, which might prevent the access of DSBs to proteins that mediate alt-NHEJ or because of sequestration of proteins through protein-protein interactions.

Consistent with this, WRN has been shown to interact with several key proteins of the c-NHEJ and alt-NHEJ pathways^9^,^12^,^13^,^33^,^34^,^47^. WRN strongly interacts with the Ku complex, which promotes c-NHEJ and inhibits alt-NHEJ. Ectopically expressed WRN stimulated c-NHEJ only in Ku70 proficient cells, demonstrating the functional importance of WRN's interaction with the Ku complex. However, WRN expression suppressed alt-NHEJ-mediated repair events in both Ku70-proficient and Ku70-deficient cells. Interestingly knockdown of Ku70 reduced WRN expression. These results suggest that WRN-deficiency might be promoting alt-NHEJ in Ku70 knockdown cells. PARP1 activity is known to promote alt-NHEJ (ref. 38), and the expression of WRN in PARP1 siRNA treated or olaparib treated cells further decreased the alt-NHEJ activity. The observed additive effect on alt-NHEJ activity could be because of lack of a promoting factor coupled with the abundance of an inhibitory factor. We previously reported that PARP1 interacts with WRN and inhibits WRN functions (ref. 50). This interaction between WRN and PARP1 may influence DNA repair pathways directly or indirectly by regulating the functions of other proteins like MRE11 and CtIP. Both WRN and PARP1 interact with the MRN complex (ref. 33, 47, 51), which is required for alt-NHEJ. PARP1 is essential for MRE11 and NBS1's recruitment to DSBs (ref. 51). Although WRN interacts with the MRN complex (ref. 33), its role in MRN recruitment kinetics and downstream effects are unclear. Our results suggest that WRN regulates alt-NHEJ by limiting the functions of MRE11 and CtIP.

Ku70/80 and MRN act as sensors of DSBs and compete for binding to DSBs; however, displacement of Ku from DSBs is essential for HR (refs 52, 53). Live-cell microscopy studies revealed that both Ku and MRN rapidly bind to DSBs, but Ku binds to the DSBs earlier than MRE11 (ref. 54). To investigate why WRN-deficient cells have increased alt-NHEJ usage and increased DNA resection, we analysed the effect of WRN's recruitment to DSBs on the recruitment dynamics of MRE11 and CtIP. We found that WRN robustly recruits to DSBs before MRE11, suggesting that WRN might affect the functions of MRE11. Interestingly, a recent study showed that WRN prevents MRE11-dependent nascent strand degradation during replication stress^55^. At DSBs, MRN together with CtIP initiates DNA end resection^41^, and both HR and alt-NHEJ share the initial steps of the resection process^22^. We here show that WRN deficiency enhances the recruitment of both MRE11 and CtIP to laser-induced DSBs and increases DNA end resection. These results suggest that WRN protects the DSBs from MRN/CtIP-mediated resection. Persistent binding of Ku at DNA ends attenuates resection^53^; accordingly, it is possible that WRN acts along with Ku to limit and regulate resection and to suppress alt-NHEJ. These findings are consistent with the models that the alt-NHEJ pathway takes over DSB repair when c-NHEJ is blocked or impaired^17^,^18^,^25^,^31^.

Defective DSB repair is associated with ageing and cellular senescence^36^,^56^. NHEJ-mediated DSB repair begins to decline with ageing and is compromised in pre-senescent and senescent cells^36^. Further, the decline in NHEJ efficiency increased the deletion length and mutagenic repair. WS is characterized by accelerated ageing features and DNA repair defects. Our results show a mechanism for aberrant NHEJ and genomic instability observed in WS and in the absence of WRN.

WS patient fibroblasts display variegated translocation mosaicism characterized by the presence of multiple, variable, predominantly stable chromosomal aberrations^3^. Cancer incidence increases exponentially with age^57^, and previous reports indicate that alt-NHEJ is highly mutagenic and drives cancer-associated chromosomal translocations and telomere fusions^25^,^27^,^28^. WS patients are at elevated risk for the incidence of sarcomas relative to what is expected in age-matched normal individuals^2^. Chromosome fusions and translocations are well documented in multiple disorders and are thought to arise because of increased microhomology-mediated end joining or alt-NHEJ activity^58^. One specific form of chromosome aberration of interest is the telomere fusions because WRN plays a role in telomere biology and telomere fusions are elevated in WS patient and Wrn-knockout mouse fibroblasts^5^,^6^. WRN interacts with TRF2 and other shelterin components where it is thought to contribute to the protection of the telomeres 59,60. Lack of TRF2 destabilizes the shelterin complex and causes telomere ends to be seen as DSBs, which effectively activates the alt-NHEJ pathway and promote telomere fusions^61^. Consistent with this model, CtIP has been shown to promote fusion of destabilized telomeres^28^. Therefore, we investigated CtIP's role in telomere fusions in the absence of WRN. Loss of either Wrn or Trf2 caused elevated levels of telomere fusions while simultaneous knockdown with CtIP abrogated telomere fusions. Thus, these results demonstrate that alt-NHEJ and CtIP promote telomere fusions in the absence of Wrn.

In summary, this work establishes the central role of WRN in pathway choice between c-NHEJ and alt-NHEJ. WRN recruits to DSBs and participates in NHEJ with its enzymatic and non-enzymatic functions. At DSBs, in association with Ku, it promotes c-NHEJ with its enzymatic functions and inhibits alt-NHEJ with its non-enzymatic functions. WRN inhibits the recruitment of MRE11 and CtIP, and hinder DSB resection and alt-NHEJ (Fig. 6c). The data presented here identifies that WRN-deficient and WS cells are prone to the mutagenic alt-NHEJ pathway and uncover a new mechanism through which WRN protects against genomic instability. It will therefore be of interest to develop strategies to inhibit alt-NHEJ pathway in WS patients to lower genomic instability and increase healthy ageing.

## Methods

### Cell culture

SRY110010 normal and SRY110011 WS lymphoblasts were provided by Dr Junko Oshima. AID-DIvA cell line used for resection assays was a generous gift from Dr Gaelle Legube (University of Toulouse, FR), and EJ2-U2OS and EJ5-U2OS cell lines were a gift from Dr Jeremy Stark (City of Hope, CA). HeLa, U2OS, AID-DIvA, EJ2-U2OS, EJ5-U2OS and CRL-2037 MEF cell lines were cultured in Dulbecco's modified Eagle's medium (Life Technologies) containing 10% fetal bovine serum (FBS). AG11395 WS and GM0637 normal fibroblasts were maintained in minimal essential media (Life Technologies) supplemented with 15% FBS, 1 × essential amino acids (Life Technologies), 1 × non-essential amino acids (Life Technologies), 1 × vitamin solution (Life Technologies). Lymphoblasts (SRY110010 and SRY 110011) were grown in RPMI-1640 supplemented with 15% FBS, 1 × L-Glutamine (Life Technologies).

### Gene knockdown and transfections

Knockdown of genes were achieved by transfecting siRNA using INTERFERin (Polyplus Transfections) or by transduction using lentivirus as mentioned before^35^. The following siRNA were used in this study, C; On Target Plus control (Dharmacon), WRN; 5′-GUGUAUAGUUACGAUGCUAG UGA -3′, CtIP (sc-37765, Santa Cruz), 53BP1; 5′-CACACAGAUUGAGGAUACG-3′. Lentivirus for transduction were prepared with pLKO.1 control shRNA, TRCN0000004899 (WRN), TRCN0000335501 (mWrn), TRCN0000071307 (mTrf2) and TRCN0000305311 (mCtip) Mission shRNA constructs by following the standard protocol. For preparing lentivirus, 5 μg of shRNA expressing plasmid, 2.5 μg of pCMV-VSV-G (Addgene) and 2.5 μg of pCMV-dR8.2 dvpr (Addgene) were mixed with 40 μL of FuGENE HD transfection reagent (Promega) in 1000 μl Opti-MEM (ThermoFisher Scientific) and transfected to 3 × 10^6^ 293T cells in 10 cm plates. 48 h post-transfection, lentivirus containing medium was harvested, filtered with 0.45 μ filter, flash frozen and stored at −80°C. For knockdown using lentivirus, 3 × 10^6^ U2OS or MEFs were seeded onto 10 cm plates, and 24 h post-seeding cells were treated with 8 μg/ml Polybrene (Sigma) and lentivirus. 48 h post transduction, shRNA expressing cells was selected and maintained in culture media containing 4 μg/ml Puromycin dihydrochloride (Sigma-Aldrich). Knockdown efficiency was tested by western blotting.

### Immunoblotting

Three days post siRNA transfection, cells were lysed with lysis buffer (50 mM Tris (pH 7.4), 150 mM NaCl, 1% Triton X-100, 1 mM EDTA and 1 mM DTT) and extracts were prepared after centrifugation at 13,000*g*, 4 °C for 10 min. Proteins were resolved in 4–15% Mini-PROTEAN TGX gels (BioRad), electroblotted to nitrocellulose membrane, and visualized using antibodies against WRN (in house), DNA-PKcs (G4; Santa Cruz), 53BP1 (C19, BD biosciences), DNA ligase IV (sc-271299, Santa cruz), Ku80 (C20; Santa Cruz), Ku70 (N3H3, Santa Cruz), CtIP (14-1, Active Motif), Artemis (N3C3, GeneTex), XLF (ab33499, Abcam), DNA ligase I (sc-20222, Santa Cruz), DNA ligase III (1F3, GeneTex), PARP1 (4C10-5, BD biosciences), XRCC1 (GTX23133, GeneTex), actin (sc-1616, Santa Cruz) and tubulin (sc-5286, Santa Cruz). Further details on the antibodies can be found in Supplementary Table 1. Uncropped images of immunoblots are shown in Supplementary figure 7.

### *In vitro* and *in vivo* DSB repair assays

Whole cell extracts for *in vitro* NHEJ assays were prepared as mentioned before^17^,^35^ from SRY110010, SRY11011 lymphoblasts and control and WRN knockdown HeLa as well as U2OS cells. *In vitro* NHEJ assays were performed as before^35^,^37^ with DNA substrates derived from *Sal*I linearized pUC18 plasmid (cohesive ends) and from 5.7 kb non-cohesive substrate from *I-Sce*I digested pSingle-tTS-plasmid (Clontech). Briefly 5 or 10 ng of cohesive and non-cohesive substrates were incubated with 20 μg of whole cell extracts in 10 μl reaction containing (20 mM HEPES (pH 7.5), 10 mM MgCl_2_, 80 mM KCl, 1 mM DTT, 1 mM ATP and 50 μM dNTP) for 1–3 h at 25 °C. The reaction was stopped with 50 mM EDTA and 80 μg ml^−1^ RNaseA at 37 °C, and deproteinized by incubating with proteinase K (2 mg ml^−1^) for 60 min at 37 °C. Ligation products were separated in a 0.7% agarose gel and stained with SYBR-Gold (Invitrogen). Fluorescence was detected with Molecular Imager Gel Doc XR system (Bio-Rad) and DNA bands were analysed with the ImageJ software (version 1.4).

*In vivo* NHEJ assays were performed with pEGFP-Pem1-Ad2, EJ2 and EJ5 report systems. For NHEJ experiments with the pEGFP-Pem1-Ad2 reporter, control and WRN knockdown U2OS cells (1 × 10^5^) were transfected with 100 ng of *Hin*dIII or *I-sce*I (New England Biologicals) cut pEGFP-Pem1-Ad2 along with 25 ng of pDsRed-Express-C1 (Clontech) using JetPrime (Polyplus Transfections). Twenty-four-hour post transfection, cells were trypsinized, harvested, resuspended in 0.5 ml phosphate-buffered saline and assayed for the expression of EGFP and DsRed by flow cytometry (Accuri C6; BD Biosciences). To measure NHEJ efficiency in EJ2 and EJ5 cells, DSBs were induced in EJ2 and EJ5 reporter cassettes by transfecting 2 μg of *I-Sce*I plasmid into 1 × 10^6^ cells using Amaxa Cell Line Nucleofector Kit V (Lonza) by following the company's protocol. For normalization, cells were transfected with 25 ng of pDsRed-Express-C1 along with *I-Sce*I plasmid. For over expression studies, 1 μg pCMV-tag4 plasmids carrying 3xFLAG tagged WT-WRN, K-WRN, X-WRN, R993A-WRN and F1037A-WRN were transfected along with *I-Sce*I and pDsRed-Express-C1 using Amaxa Cell Line Nucleofector Kit V. For knockdown experiments, 24 h post siRNA transfection, 1 × 10^5^ cells were transfected with *I-Sce*I and pDsRed-Express-C1. Four days after *I-Sce*I transfection, cells were harvested and analysed by flow cytometry for the expression of GFP and DsRed. Flow cytometry data were collected from 10,000 to 50,000 cells. For inhibition of PARP1 activity, EJ2 cells transfected with plasmids expressing 3xFLAG-WT-WRN, *I-Sce*I and DsRed were treated with 5 μM olaparib for 4 days.

### Junctional sequence analysis

End products from the *in vitro* end-joining assays, with 5.7 kb substrate with non-cohesive ends, were purified by phenol-chloroform extraction and ethanol precipitation. End-joined regions were amplified by polymerase chain reaction using HotStarTaq DNA polymerase (Qiagen) and primers (5′-ATGC**AAGCTT**CCTTTATTACCCAGAAGTCAG ATGC-3′ and 5′-ATGC**TCTAGA**GTAAACTCGCCCAGAAGCTAGG-3′; substrate sequences are underlined and restriction endonuclease sites are highlighted in bold). PCR was carried out in a 50-μl reaction mixture for 35 cycles (denaturation, 94 °C; annealing, 62 °C; extension, 72 °C) and amplified end-joined products were purified by ethanol precipitation, digested with *Xba*I and *Hin*dIII (New England BioLabs), and cloned into the pUC18 vector using HB101 competent cells. DNA from at least 30 clones for each sample from three independent experiments was sequenced using the M13F primer.

### DNA end resection assays

*In vivo* 5′ end resection at an *Asi*SI endonuclease-induced DSB was measured in AID-DIvA U2OS cells^43^. The extent of resection at DSB site created on chromosome 1 was measured by amplifying ssDNA using TaqMan qPCR as previously described^44^. Briefly, AID-DIvA cells were transfected with C, WRN, CtIP and Ku70 siRNA using INTERFERin and two days post transfection the cells were transferred to 10 cm plates and 24 h later the cells were treated with 4-OHT (Sigma) for 4 h. Genomic DNA was isolated using Blood & Cell Culture DNA mini kit (Qiagen). RNaseH (NEB) treated genomic DNA of 3 μg was mock digested or digested with *Bsr*GI or *Hin*dIII (NEB) at 37 °C overnight. To quantify the extent of resection, 18 ng of mock digested and restriction enzyme digested samples were amplified by qPCR using specific Taqman probes^44^. The ssDNA per cent was calculated from the change in Ct values of mock and restriction enzyme digested DNA using the following equation: ssdNA%=1/(2^^(Δ*Ct*−1)+0.5^) × 100. At least three biological repeats were performed and data were presented with mean±s.e.m.

### Measurement of chromatin-bound RPA

DNA end resection was investigated by measuring chromatin-bound RPA as previously described^38^,^45^. Briefly, siRNA (control and WRN) transfected U2OS cells were treated with 1 μM camptothecin (CPT) for 1 h. Harvested cells were treated with 1 ml of 0.2% Triton X-100 in PBS on ice for 7 min and washed with 1 ml Perm/Wash buffer (BD Biosciences) and fixed with 300 μl of Cytofix/Cytoperm buffer (BD Biosciences) for 15 min. Following fixation, cells were washed with Perm/Wash buffer for 30 min and treated with 1 μg ml^−1^ RPA antibody (NA-18, EMD Millipore) in BD Perm/Wash buffer for 1 h at 37 °C and later with 1:500 goat anti-mouse Alexa Fluor 488 (Life Technologies) at 37 °C for 30 min. Immunostained cells were washed with Perm/Wash buffer and suspended in 0.3 ml PBS containing 10 μg/ml propidium iodide (Sigma), 250 μg/ml RNase A (Thermo Fisher) and 0.02% sodium azide (Sigma) for 15 min at 37 °C. Chromatin-bound RPA-positive cells were detected with Accuri C6 Flow Cytometer (BD Biosciences) and analysed with FlowJo v10 (FlowJo). The data represents average of three biological experiments with SEM. *P* values were calculated with *Student t*-Test.

### Microirradiation, Immunofluorescence and microscopy

DSBs in U2OS and AG11395 cells were generated with a Stanford Research Systems (SRS) NL100 nitrogen MicroPoint system (Photonics Instruments) equipped to a Nikon Eclipse TE2000 spinning disk confocal microscope (Nikon Instruments Inc.). Site specific DSBs in 0.25 × 3 μM or 1 × 10 μM tracks were induced with 435 nm laser regulated through Volocity software 6.3 (Perkin-Elmer). The microscope was supported with temperature and CO_2_–regulated incubation chamber. Following microirradiation, cells were washed with PBS, fixed (3.7% formaldehyde in PBS for 10 min), permeabilized (0.1% Triton-X 100 in PBS for 10 min) and blocked with 5% FBS for 1 h at room temperature. G1 and S/G2 phase cells were detected by staining the cells first with cyclin A2 antibody (ab16726, Abcam) for 1 h followed by donkey anti-mouse Alexa488 antibody for 1 h at 37 °C. Following cyclin A2 staining, the endogenous proteins (WRN, MRE11, CtIP, DNA ligase I and DNA ligase III) recruited to the DSBs were detected by incubating the cells with primary antibodies (γH2AX, JBW301 Millipore or sc-101696, Santa Cruz; WRN, in house antibody; MRE11, ab214, Abcam; CtIP, Active Motif; DNA ligase I, Santa Cruz; DNA ligase III, GeneTex) for 2 h and then with secondary antibodies (donkey anti-mouse Alexa488, donkey anti-goat Alexa 594, donkey anti-rabbit Alexa 647, Life Technologies) for 1 h at 37 °C in a humidified chamber. Cells were washed four times with 0.1% Tween-20 in PBS for 40 min after incubation with antibodies, and mounted in ProLong Gold antifade mounting media with DAPI (Life Technologies). The images were captured with a confocal microscope and analysed with Volocity software. For assessing real-time recruitment dynamics of MRE11 and CtIP in the presence and absence of WRN, AG11395 cells were transfected with plasmids expressing YFP-MRE11, GFP-CtIP and mCherry-WRN using jetPRIME transfection reagent. Following the laser induced DSBs, the recruitment of fluorescent labelled proteins were recorded at 10–20 s interval for 5 min with a CCD camera (Hamamatsu) and Volocity software.

### Preparation of metaphase spreads and telomere FISH

CRL-2037 MEFs (1 × 10^6^) were transduced with lentivirus carrying control shRNA or shRNA against *Wrn*, *Trf2* and or *Ctip*. After 48 h post transduction, the cells were provided with fresh media containing 4 μg ml^−1^ puromycin (Sigma) for 5–15 days. Cells were treated with 0.2 μg ml^−1^ colcemid (Life Techonologies) for 6 h, harvested and swollen in pre-warmed 75 mM KCl at 37 °C for 30 min. Swollen cells were fixed in freshly prepared 3:1 mix of methanol:glacial acetic acid and dropped onto Superfrost Plus micro slides (VWR international). The slides were air dried, heated at 42 °C for 1 min and overnight stored under dark condition. Next day, cells were hydrated in PBS, fixed with 4% formaldehyde for 2 min and treated with pepsin (1 mg ml^−1^) for 10 min at 37 °C and washed in PBS twice. Cells were dehydrated in ethanol series (70%, 95% and 100%) for 5 min and air dried. 40 μl of telomere probe mix (1 μM Cy3-conjugated PNA(TTAGGG)_3_, 10 mM Tris–HCl pH 7.2, 70% deionized formamide, 0.5% blocking reagent (100 mM maleic acid,150 mM NaCl, pH 7.5)) was dropped onto slide and sealed with a coverslip. Following the denaturation on a hotplate at 80 °C for 10 min, the slides were incubated at 37 °C for 2 h in dark humidified chamber. Slides were washed twice with wash buffer 1 (10 mM Tris–HCl pH 7.2, 70% formamide, 0.1% BSA) and then thrice with wash buffer 2 (100 mM Tris–HCl pH 7.2, 150 mM NaCl, 0.08% Tween-20). After air drying at room temperature, slides were mounted with ProLong Gold with DAPI (Life Technologies). Micrographs of mitotic chromosomes were taken with AxioVision software using Zeiss Axiovert 200 M fluorescence microscopy equipped with AxioCam HRM camera.

### Data availability

The authors declare that all data supporting the findings of this study are available within the article and its Supplementary files or from the authors on a reasonable request.

## Additional information

**How to cite this article:** Shamanna, R. A. *et al*. WRN regulates pathway choice between classical and alternative non-homologous end joining. *Nat. Commun.* **7,** 13785 doi: 10.1038/ncomms13785 (2016).

**Publisher's note**: Springer Nature remains neutral with regard to jurisdictional claims in published maps and institutional affiliations.

## Supplementary Material

Supplementary Information Supplementary Figures 1-7 and Supplementary Table 1.

Supplementary Movie 1 Recruitment of mCherry-WRN and YFP-MRE11 to laser-induced DSBs.

Supplementary Movie 2 Recruitment of YFP-MRE11 in WRN-deficient AG11395 cells.

Supplementary Movie 3 SRecruitment of YFP-MRE11 in mCherry-WRN expressing AG11395 WS cells.

Supplementary Movie 4 Recruitment of GFP-CtIP in AG11395 WS cells.

Supplementary Movie 5 Recruitment of GFP-CtIP in mCherry-WRN expressing AG11395 WS cells.
