# Supplementary material for: WRN regulates pathway choice between classical and alternative non-homologous end joining
Source: Nat Commun. 2016 Dec 6;7:13785. doi: 10.1038/ncomms13785 (PMC5150655; doi:10.1038/ncomms13785)
Supplement: Supplementary Information — Supplementary Figures 1-7 and Supplementary Table 1. [file ncomms13785-s1.pdf]

A

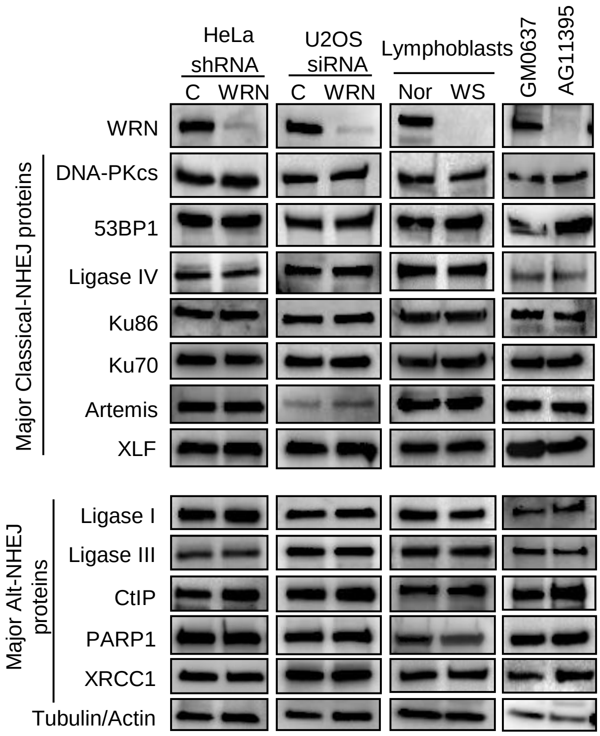

B

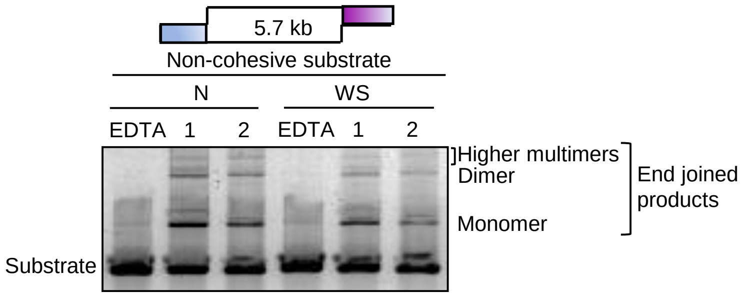

Supplementary figure 1. (A) Expression of DNA repair proteins in WRN deficient cells. Extracts from shRNA/siRNA-treated HeLa and U2OS cells, lymphoblasts (normal [N]; SRY110010, WS; SRY110011) and fibroblasts (normal; GM0637, WS; AG11395) were immunoblotted for indicated DNA repair proteins. (B) WS cells show NHEJ deficiency *in vitro.* Agarose gel showing products from NHEJ reaction with 5.7-kb non-cohesive DNA substrate and cell extracts prepared from normal (N) and WS lymphoblasts.

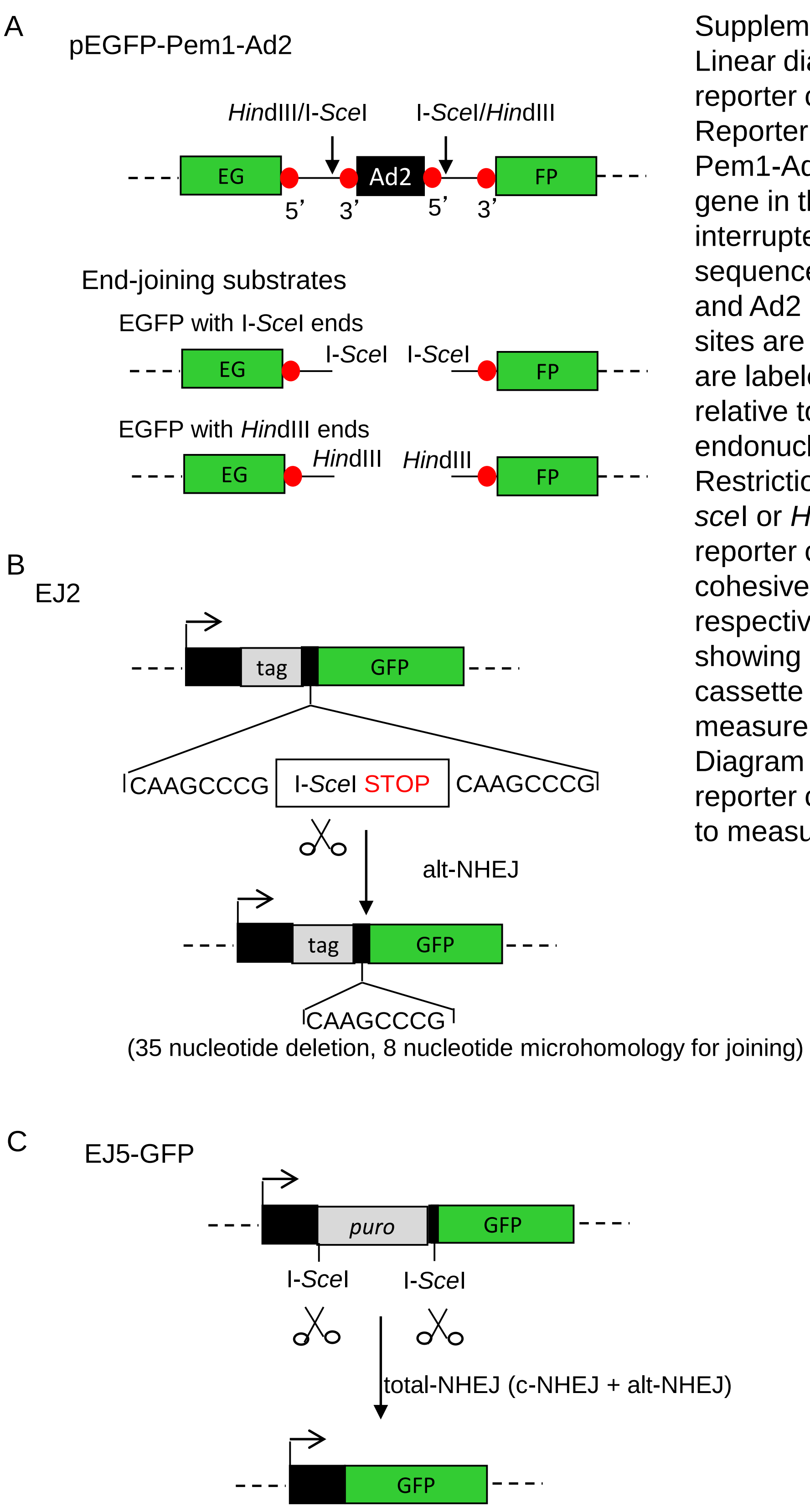

Supplementary figure 2. Linear diagrams of NHEJ reporter cassettes. (A) Reporter cassette of pEGFP-Pem1-Ad2 plasmid. EGFP gene in the plasmid is interrupted by a stuffer sequence containing Pem1 and Ad2 intron. Endonuclease sites are marked. Splice sites are labeled (red circles) relative to the intron and endonuclease sites. Restriction digestion with *I-sceI* or *Hind*III regenerates reporter cassettes with non-cohesive and cohesive ends, respectively. (B) Diagram showing GFP reporter cassette in EJ2 cells to measure alt-NHEJ. (C) Diagram showing GFP reporter cassette in EJ5 cells to measure total NHEJ.

Supplementary figure 3

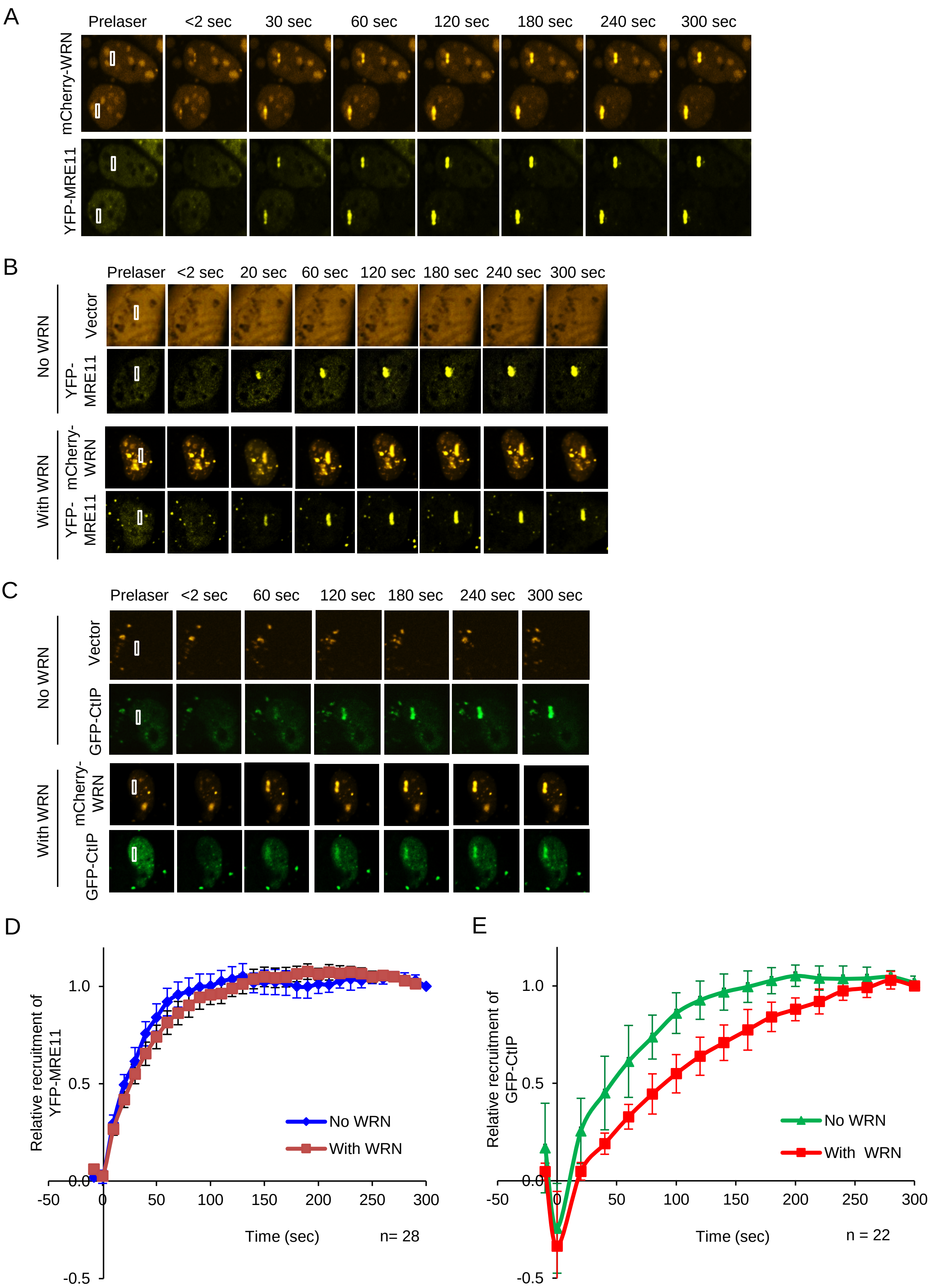

Supplementary figure 3. WRN deficiency increases recruitment of MRE11 and CtIP to DSBs. (A) Representative images showing recruitment of mCherry-WRN and YFP-MRE11 to microirradiated laser tracks in U2OS cells. (B) Micrographs showing YFP-MRE11 recruitment to laser-induced DSB tracks in AG1135 WS cells transfected with vector (No WRN) and mCherry-WRN (with WRN). (C) Micrographs showing GFP-CtIP recruitment to DSBs in WS cells, AG11395, in the absence and presence of WRN. (D) Graph showing YFP-MRE11 recruitment kinetics to DSBs in the presence and absence of WRN. (E) GFP-CtIP recruitment kinetics in AG11395 cells expressing with and without mCherry-WRN. White boxes represent microirradiation tracks.

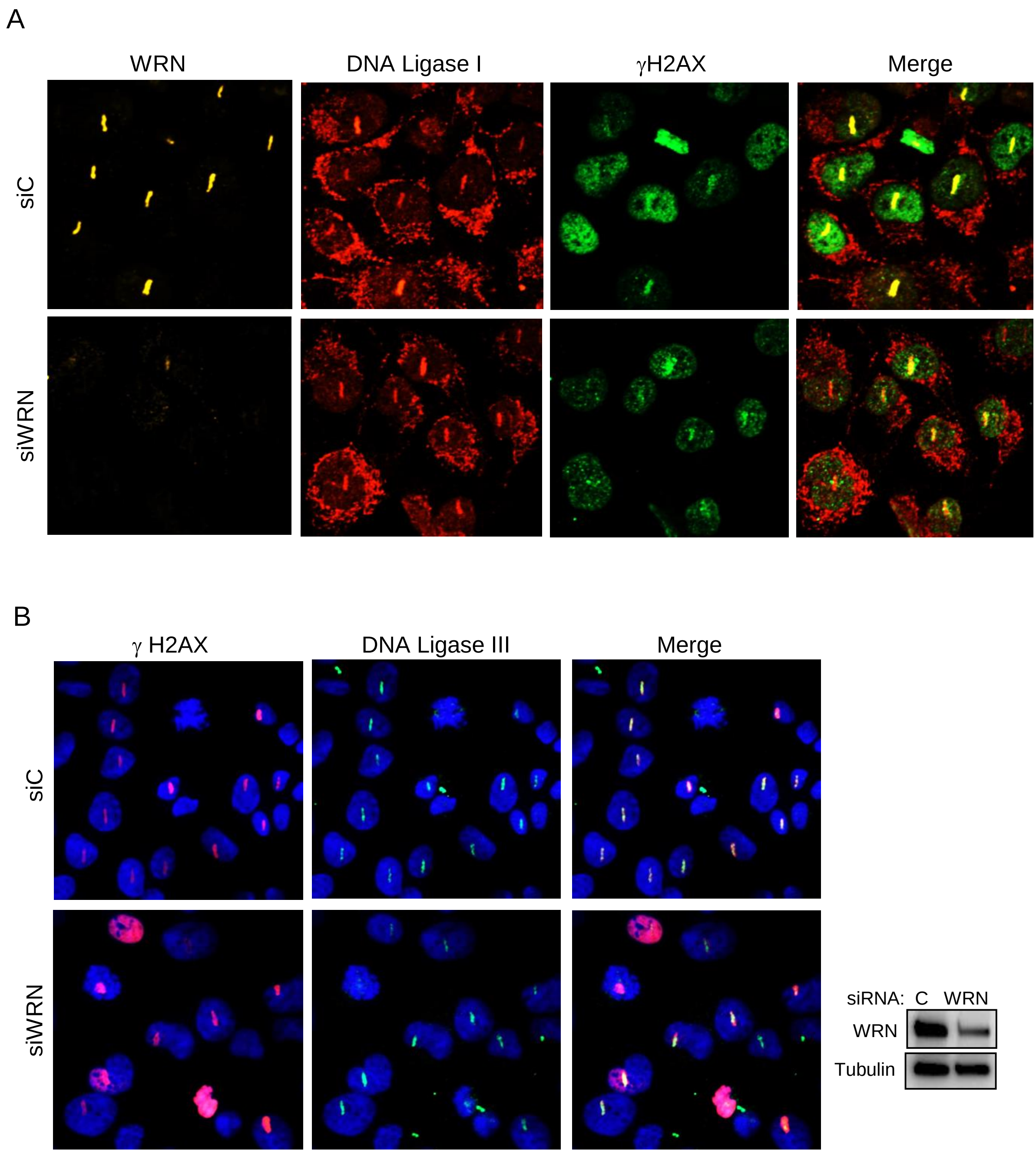

Supplementary figure 4. WRN deficiency does not affect the recruitment of DNA ligase I and ligase III to DSBs. (A) Recruitment of DNA ligase I to DSBs in control and WRN siRNA transfected U2OS cells. DSBs were induced by microirradiation and cells were stained with WRN, DNA ligase I and γH2A.X antibodies. (B) Recruitment of DNA ligase III to DSBs in siRNA transfected U2OS cells. DSB and immunostaining with indicated antibodies were performed as in panel A. Immunoblots represent WRN knockdown levels. siC; control siRNA, siWRN; WRN siRNA.

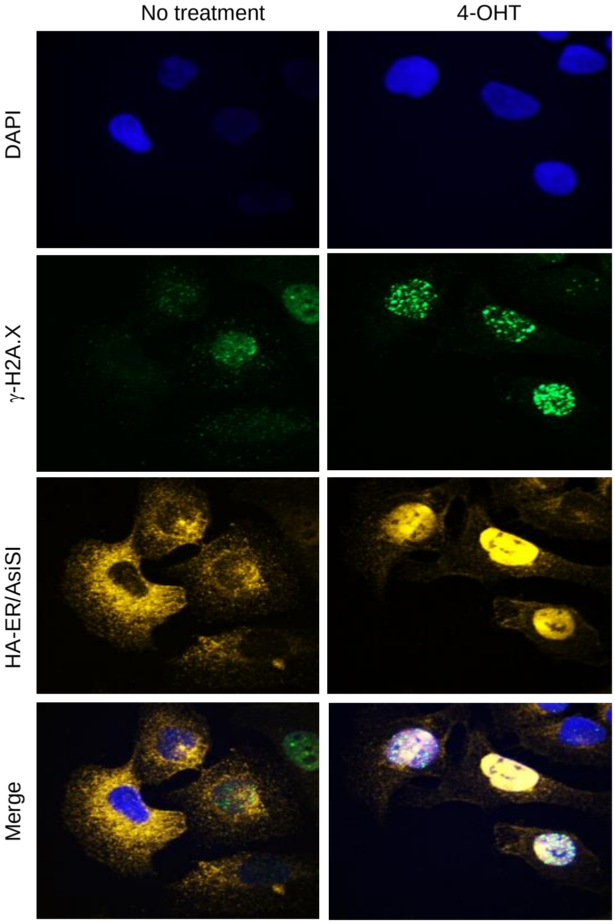

Supplementary figure 5. Induction of DSBs in AID-DIvA cells. Cells were treated with 4-OHT (4-hydroxy tamoxifen) for 4h and immunostained with HA and γH2A.X antibodies to detect HA-ER tagged *Asi*SI and phosphorylated (S139) H2A.X. DSBs induction is indicated by γH2A.X foci.

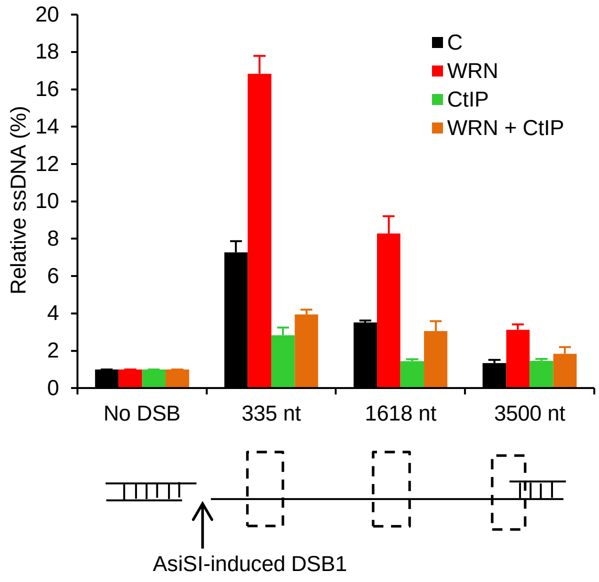

Supplementary figure 6. Quantitation of 5'end resection in AID-DIvA cells transfected with control, WRN and CtIP siRNA. Graph represents data from two independent experiments. Error bars represent SEM.

Supplementary figure 7 Uncropped blots

Figure 2A

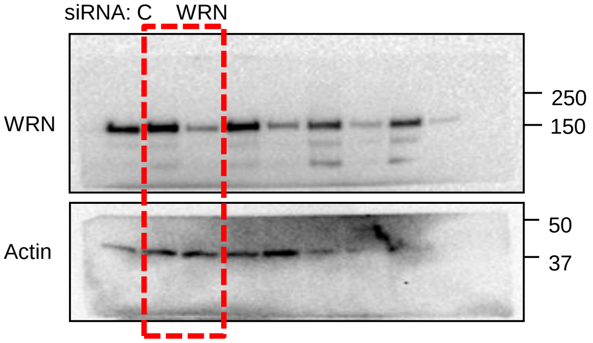

Figure 2B

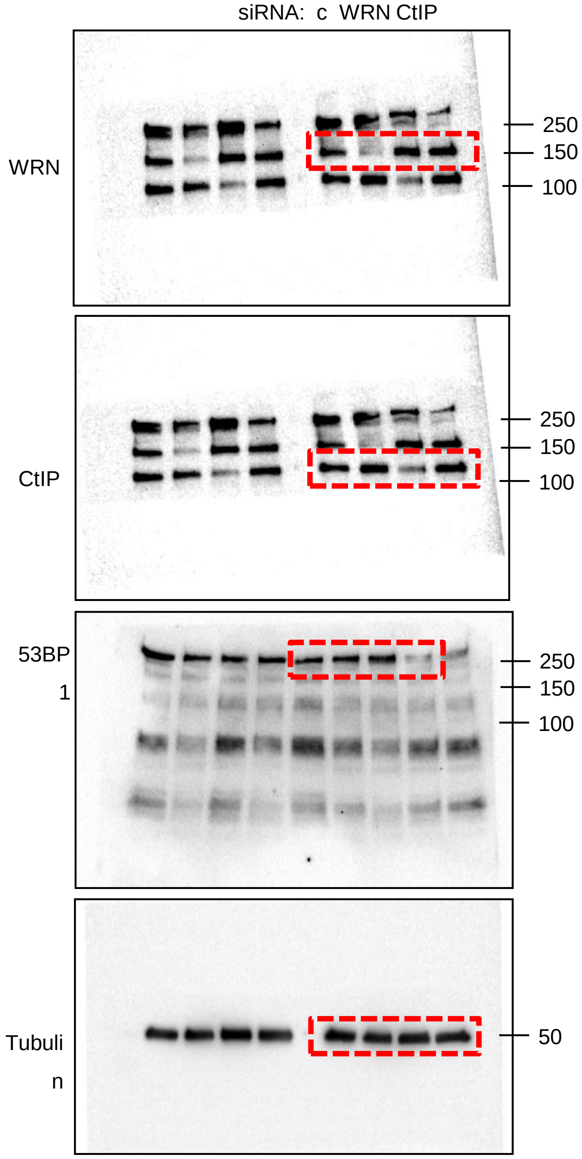

Figure 2C

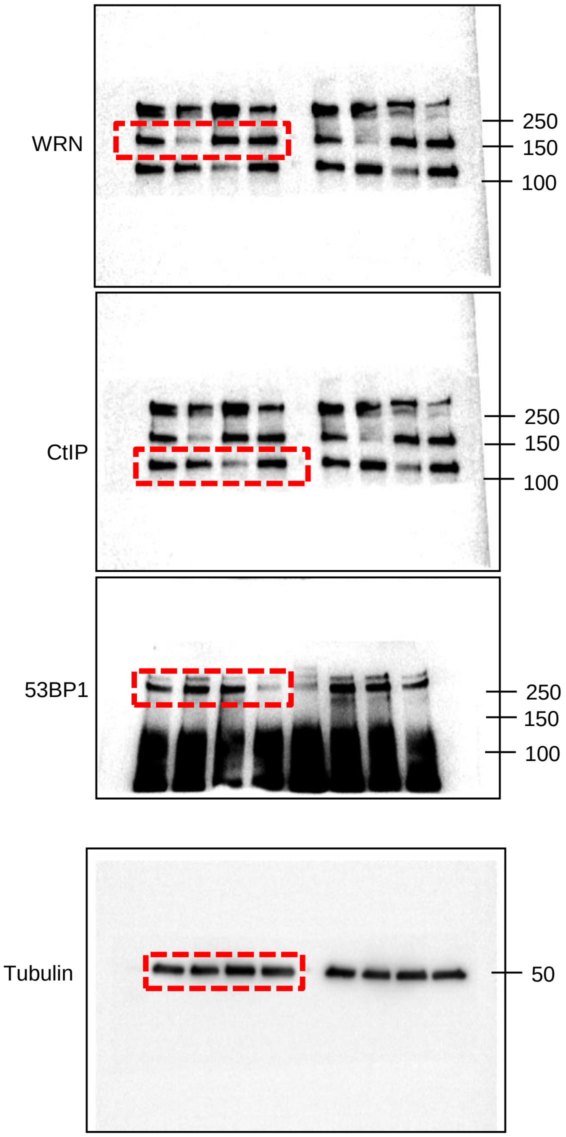

Figure 2D

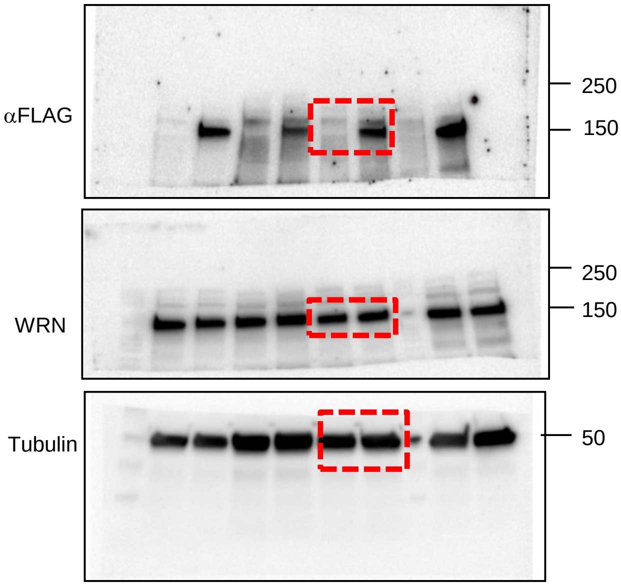

Figure 2E

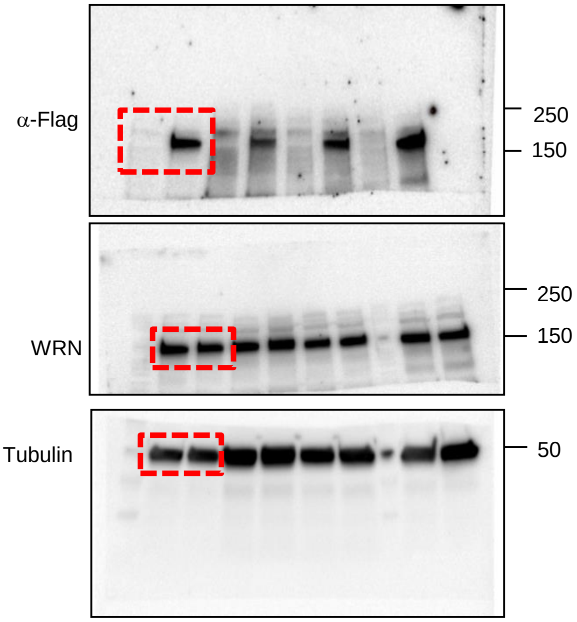

Supplementary figure 7 ---- continued

Uncropped blots

Figure 3C

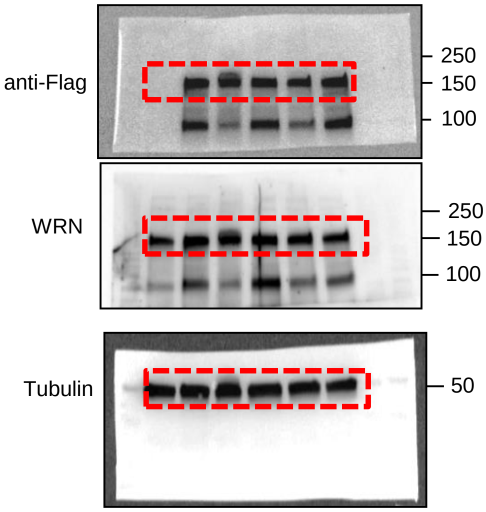

Figure 3D

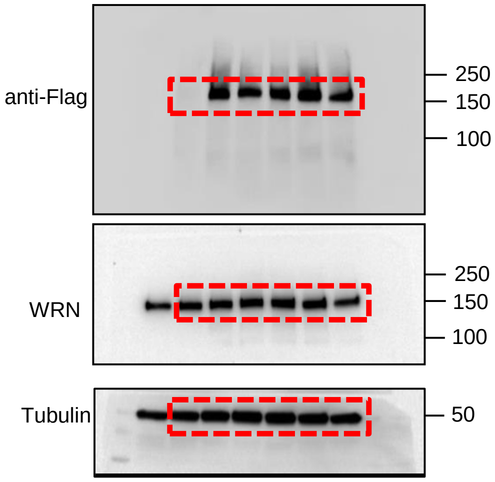

Figure 3E

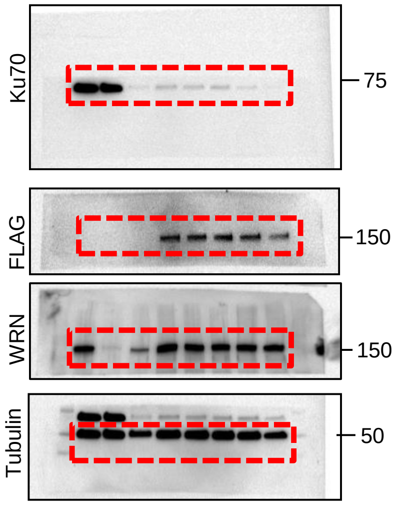

Figure 3F

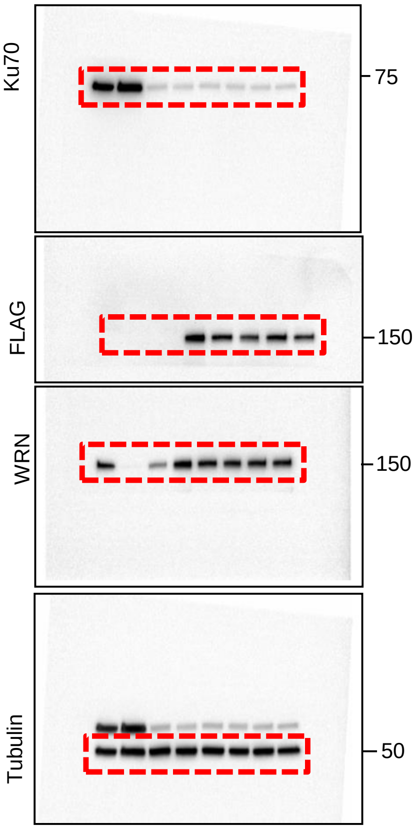

Figure 3G

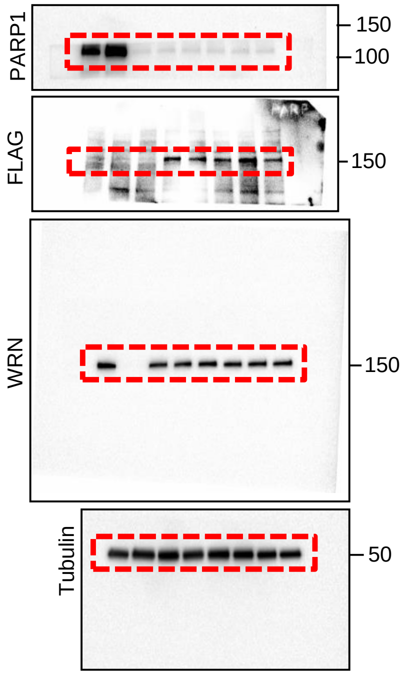

Supplementary figure 7 ---- continued

Uncropped blots

Figure 5A

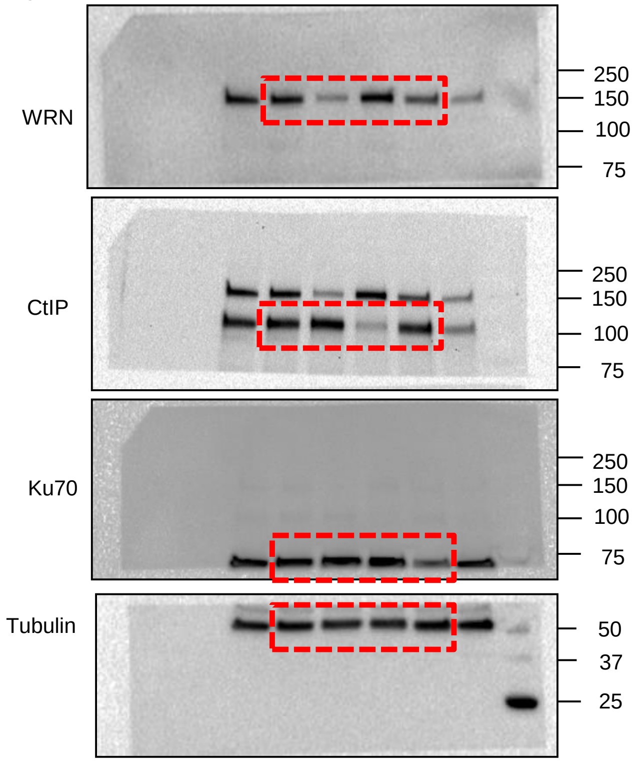

Figure 5B

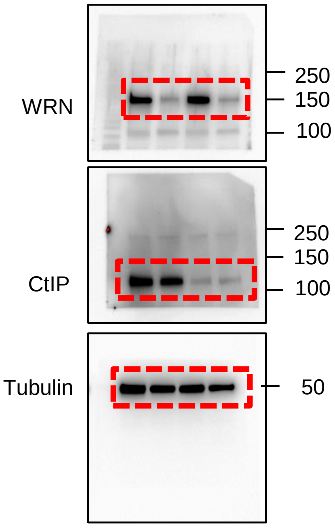

Figure 6B

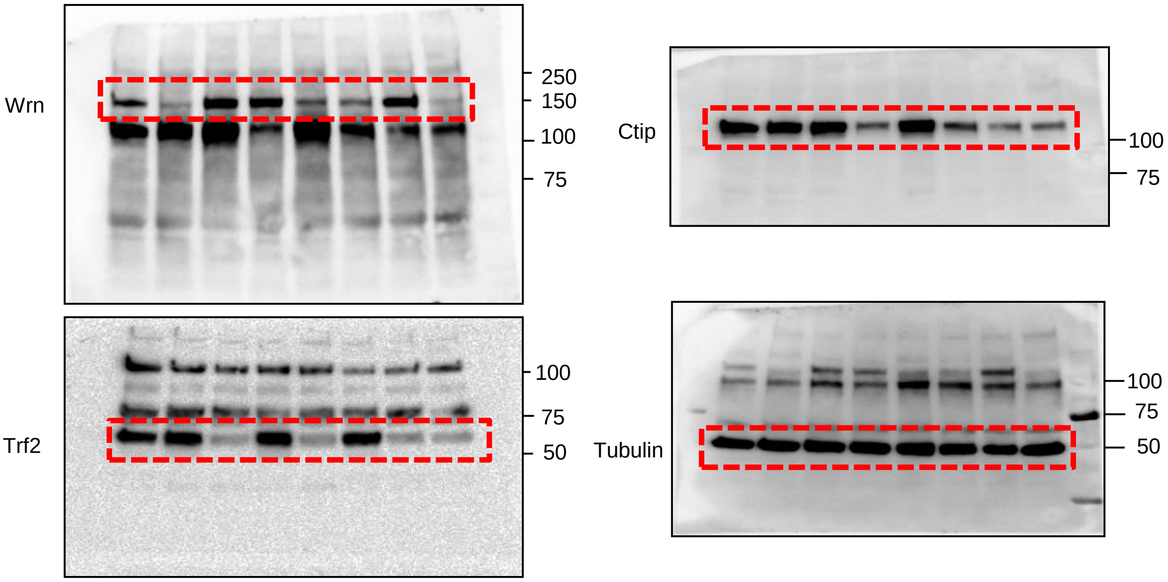

Supplementary table 1. List of antibodies used in this study

Antibody	Cat. Number	Vendor	Dilution	Usage
DNA-PKcs	G4: sc-5282	Santa Cruz	1:2000	WB
DNA ligase IV	sc-271299	Santa cruz	1:1000	WB
Ku80	C20	Santa Cruz	1:1000	WB
Ku70	N3H3	Santa Cruz	1:3000	WB
DNA ligase I	sc-20222	Santa Cruz	1: 1000<br>1: 100	WB<br>IF
actin	sc-1616	Santa Cruz	1:2000	WB
tubulin	sc-5286	Santa Cruz	1: 2000	WB
CtIP	14-1	Active Motif	1:1000<br>1:100	WB,<br>IF
Artemis	N3C3	GeneTex	1:1000	WB
XLF	ab33499	Abcam	1:1000	WB
DNA ligase III	1F3	GeneTex	1: 1000<br>1:50	WB<br>IF
PARP1	4C10-5	BD biosciences	1: 1000	WB
XRCC1	GTX23133	GeneTex	1: 1000	WB
53BP1	C19	BD biosciences	1: 1000	WB
RPA32	NA-18	EMD Millipore	1:25	Flow cytometry
MRE11	Ab214	Abcam	1:1000<br>1:50	WB<br>IF
γH2AX	JBW301<br>sc-101696	Millipore<br>SantaCruz	1:2000<br>1:3000	IF<br>IF
CtIP	14-1	Active Motif	1:1000<br>1:50	WB<br>IF
WRN	H300	Santa Cruz	1:1000<br>1:50	WB<br>IF
Trf2	NB110-57130	Novus Biologics	1:1000	WB
